# Supplementary figures and images for: From fragmentation to resilience: Connectivity and habitat diversity as drivers of fish persistence in California watersheds
Source: PLoS One. 2025 Dec 23;20(12):e0339212. doi: 10.1371/journal.pone.0339212 (PMC12725570; doi:10.1371/journal.pone.0339212)

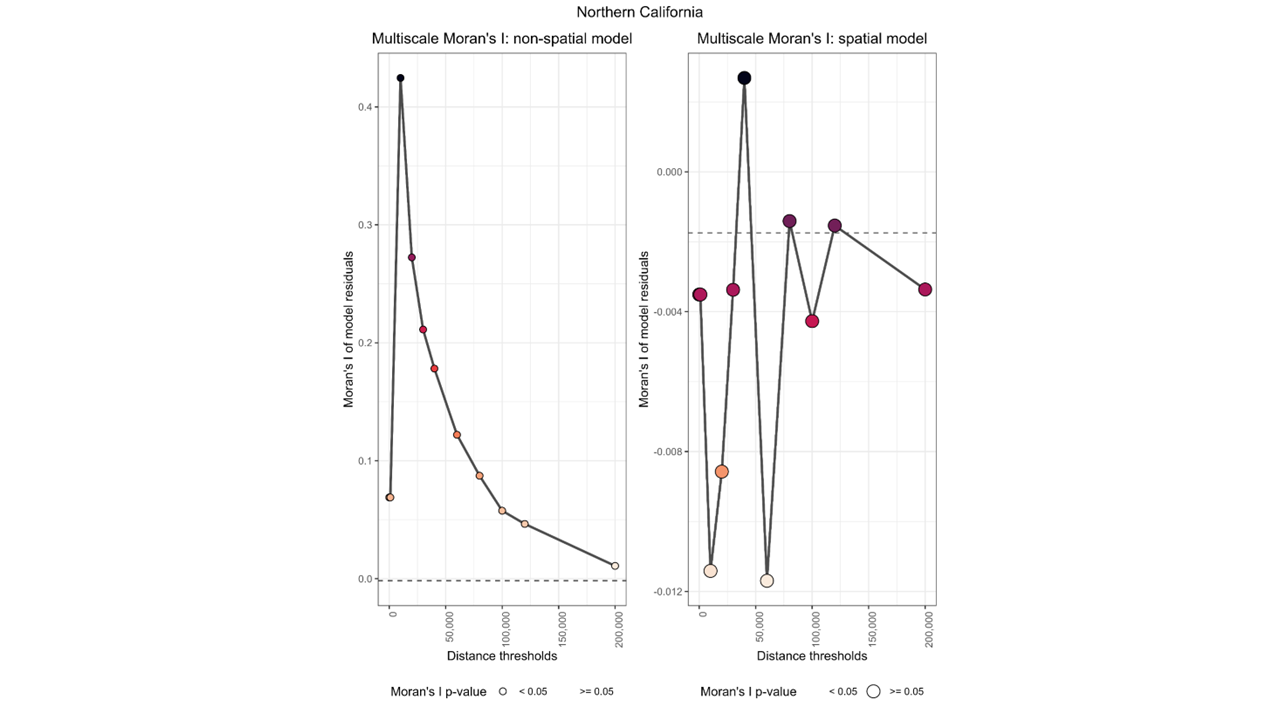

Supplement: S1 Fig — Moran’s I plots of the residuals for distance thresholds used in the non-spatial RF (first panel) and the spatial RF (second panel) for the Northern California ecoregion. P-values greater than 0.05 are considered non-significant for spatial autocorrelation and are found in the spatial RF. (TIF) [file pone.0339212.s001.tif]

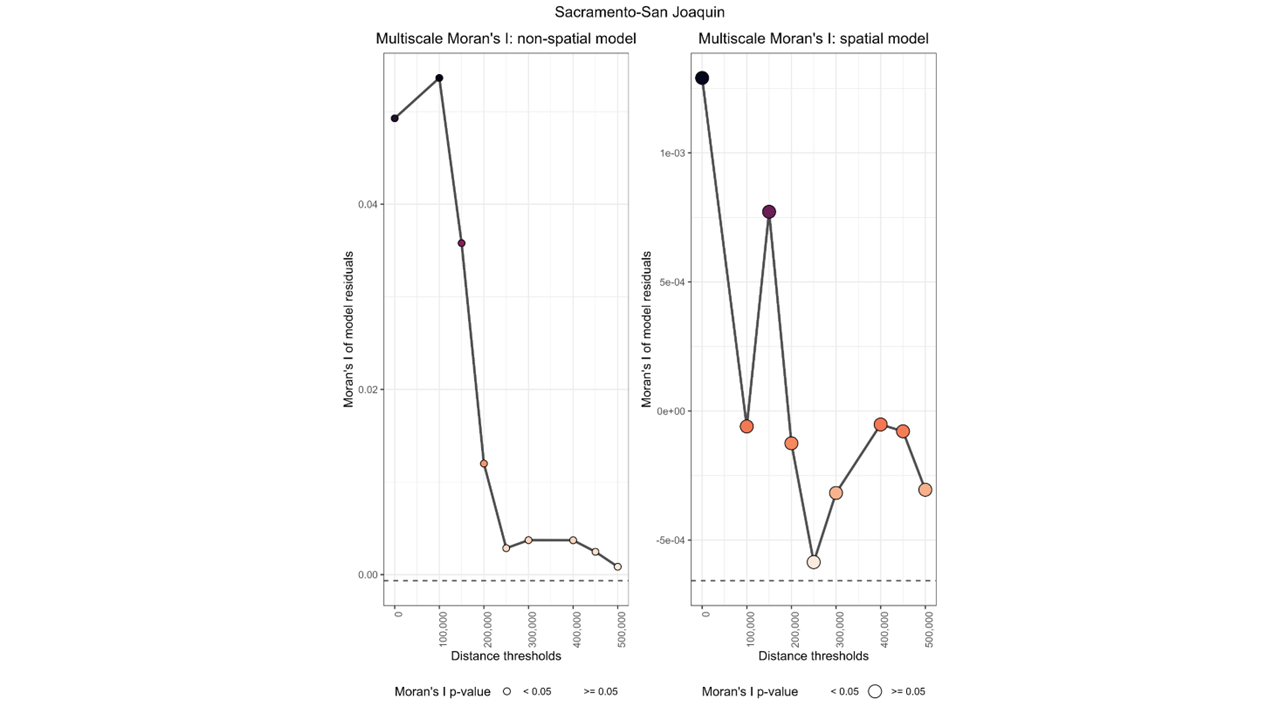

Supplement: S2 Fig — Moran’s I plots of the residuals for distance thresholds used in the non-spatial RF (first panel) and the spatial RF (second panel) for the Sacramento-San Joaquin ecoregion. P-values greater than 0.05 are considered non-significant for spatial autocorrelation and are found in the spatial RF. (TIF) [file pone.0339212.s002.tif]

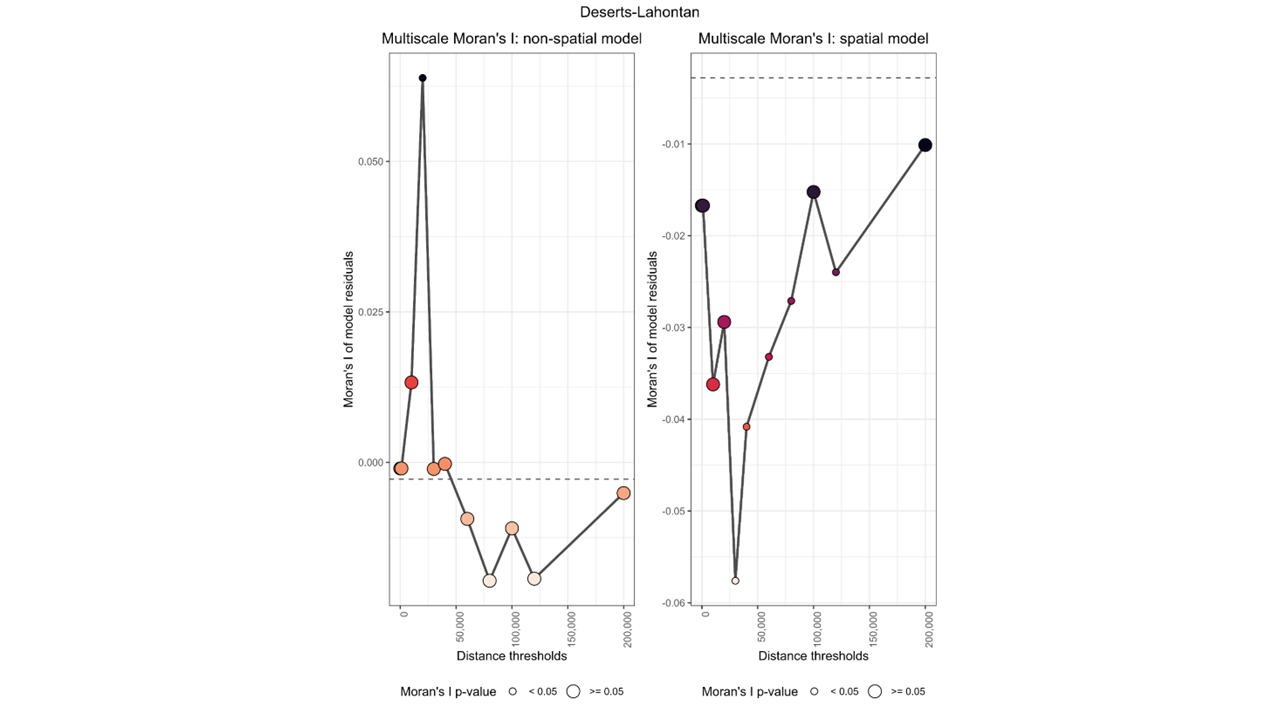

Supplement: S3 Fig — Moran’s I plots of the residuals for distance thresholds used in the non-spatial RF (first panel) and the spatial RF (second panel) for the Deserts-Lahontan ecoregion. P-values greater than 0.05 are considered non-significant for spatial autocorrelation and are found in both the non-spatial and spatial RF. (TIF) [file pone.0339212.s003.tif]

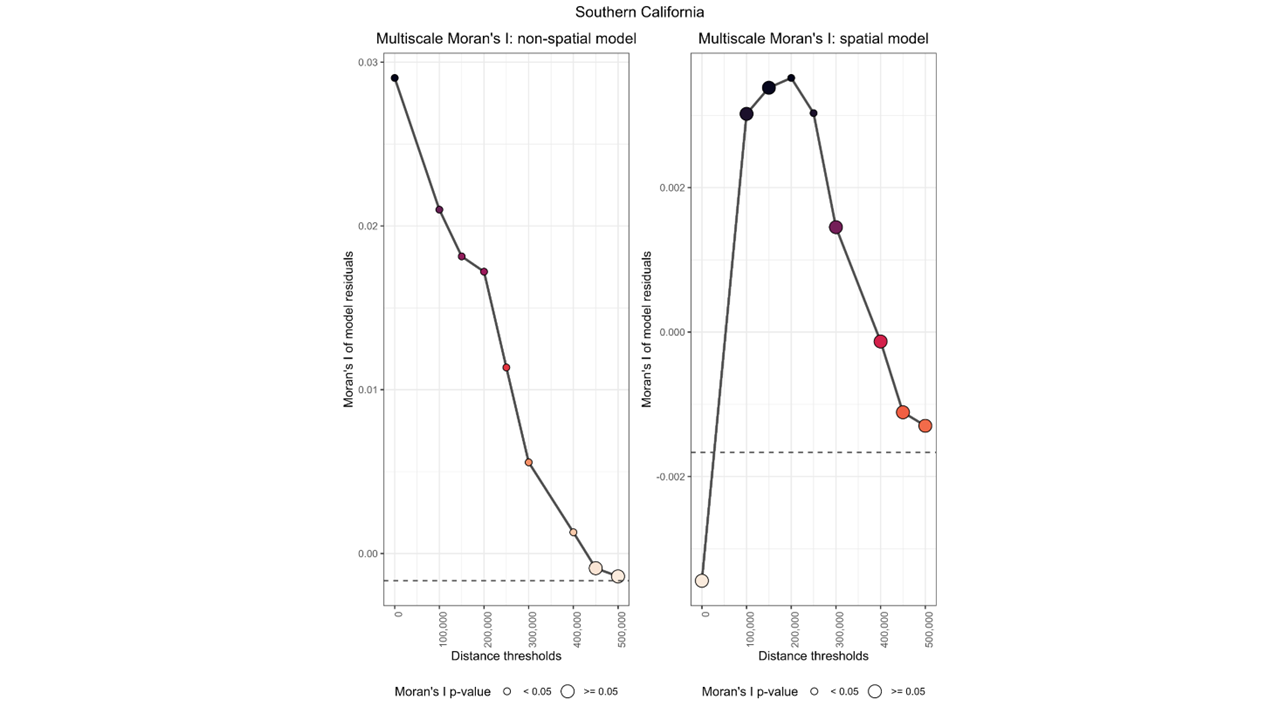

Supplement: S4 Fig — Moran’s I plots of the residuals for distance thresholds used in the non-spatial RF (first panel) and the spatial RF (second panel) for the Southern California ecoregion. P-values greater than 0.05 are considered non-significant for spatial autocorrelation and are found in the spatial RF. (TIF) [file pone.0339212.s004.tif]

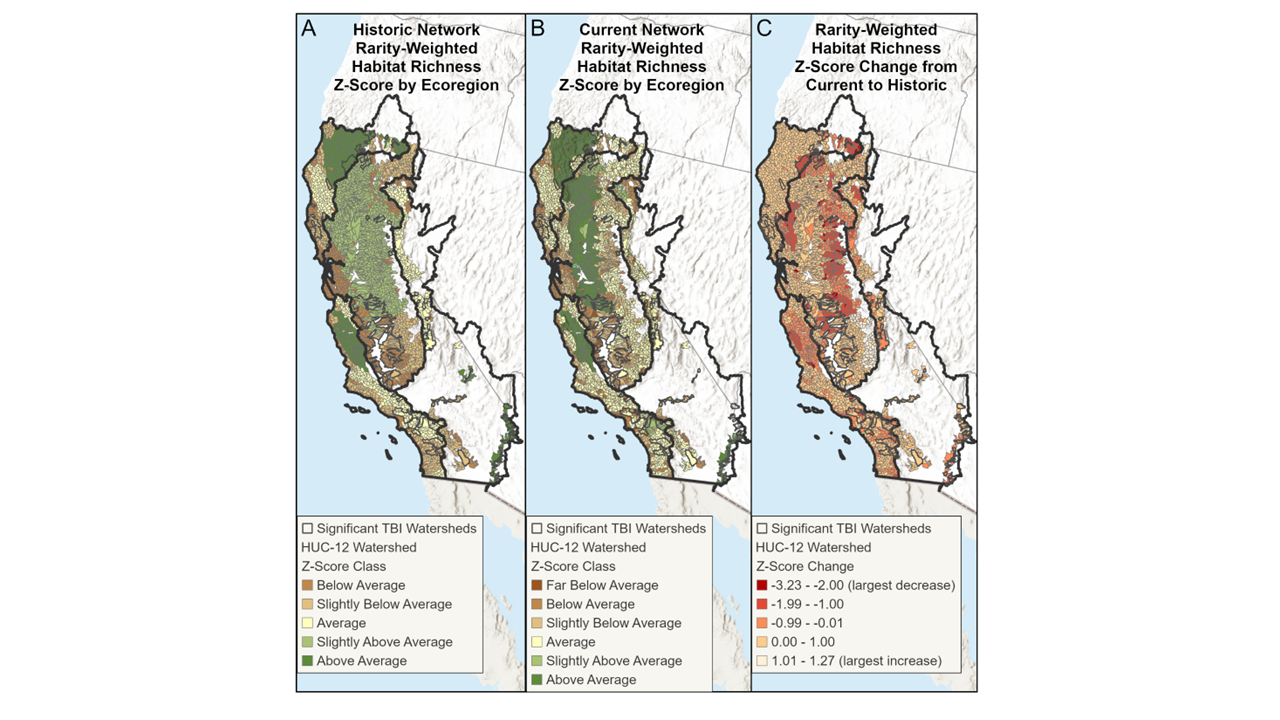

Supplement: S5 Fig — The rarity-weighted habitat richness of historic (A) and current networks (B) for HUC-12 watersheds is shown by z-score classes (Table S-5) relative to California freshwater ecoregions. Z-scores were used to facilitate comparison of the richness values for the two time periods but were not a perfect solution as discussed in the main text. Panel C shows the change in z-scores from current to historic networks where negative values indicate lower rarity-weighted richness in the current network. World Hillshade is used in the figure’s background (Sources: Esri, Airbus DS, USGS, NGA, NASA, CGIAR, N Robinson, NCEAS, NLS, OS, NMA, Geodatastyrelsen, Rijkswaterstaat, GSA, Geoland, FEMA, Intermap, and the GIS User Community). (TIF) [file pone.0339212.s005.tif]
