# Supplementary material for: From fragmentation to resilience: Connectivity and habitat diversity as drivers of fish persistence in California watersheds
Source: PLoS One. 2025 Dec 23;20(12):e0339212. doi: 10.1371/journal.pone.0339212 (PMC12725570; doi:10.1371/journal.pone.0339212)
Supplement: S1 Table — Categories used to classify stream reaches based on gradient, valley confinement, and flow regime. (DOCX) [file pone.0339212.s006.docx]

| **Layer** | **Class** | **Reach-scale criteria** | **Criteria source** |
| --- | --- | --- | --- |
| Drainage area | Headwaters | 2.5 – 10 km² | McManamay et al. 2018 |
|  | Cree  k | 10 – 100 km² | McManamay et al. 2018 |
|  | Small River | 100 – 500 km² | McManamay et al. 2018 |
|  | Medium River | 500 – 2500 km² | McManamay et al. 2018 |
|  | Mainstem | 2500 – 10000 km² | McManamay et al. 2018 |
|  | Large River | 10000 – 25000 km² | McManamay et al. 2018 |
|  | Great River | > 25000 km² | McManamay et al. 2018 |
| Water bodies | Ocean or estuary | Is an ocean or estuary | - |
|  | Natural Lake | Is a natural lake | - |
|  | Artificial Lake | Is an artificial lake | - |
| Gradient | Very Low | < 0. 1% | McManamay & DeRolph 2019 |
|  | Low | 0. 1 – 0. 5% | McManamay & DeRolph 2019 |
|  | Moderate | 0. 5 – 2% | McManamay & DeRolph 2019 |
|  | Moderate High | 2 - 4% | McManamay & DeRolph 2019 |
|  | High | 4 – 10% | McManamay & DeRolph 2019 |
|  | Steep | > 10% | McManamay & DeRolph 2019 |
| Temperature | Very Cold | < 10°C | McManamay & DeRolph 2019 |
|  | Cold | 10 – 15°C | McManamay & DeRolph 2019 |
|  | Cool-cold | 15 - 18°C | McManamay & DeRolph 2019 |
|  | Cool | 18 - 21°C | McManamay & DeRolph 2019 |
|  | Cool-warm | 21 - 24°C | McManamay & DeRolph 2019 |
|  | Warm | > 24°C | McManamay & DeRolph 2019 |
| Confinement | Unconfined | - | McManamay & DeRolph 2019 |
|  | Moderately confined | - | McManamay & DeRolph 2019 |
|  | Confined | - | McManamay & DeRolph 2019 |
| Flow regime | Snowmelt | - | McManamay & DeRolph 2019 |
|  | Stable baseflow | - | McManamay & DeRolph 2019 |
|  | Intermittent | - | McManamay & DeRolph 2019 |
|  | Snowmelt (high elevation) | - | McManamay & DeRolph 2019 |
|  | Intermittent SW | - | McManamay & DeRolph 2019 |
|  | Western runoff | - | McManamay & DeRolph 2019 |
