# Supplementary material for: From fragmentation to resilience: Connectivity and habitat diversity as drivers of fish persistence in California watersheds
Source: PLoS One. 2025 Dec 23;20(12):e0339212. doi: 10.1371/journal.pone.0339212 (PMC12725570; doi:10.1371/journal.pone.0339212)
Supplement: S2 Table — List of the 129 native freshwater fish species used to conduct this analysis. (DOCX) [file pone.0339212.s007.docx]

| **Scientific Name** | **Common Name** | **Range** |
| --- | --- | --- |
| Cyprinodon nevadensis amargosae | Amargosa River pupfish | narrow |
| Cyprinodon nevadensis nevadensis | Saratoga Springs pupfish | narrow |
| Cyprinodon nevadensis shoshone | Shoshone pupfish | narrow |
| Cyprinodon salinus milleri | Cottonball Marsh pupfish | narrow |
| Cyprinodon salinus salinus | Salt Creek pupfish | narrow |
| Gasterosteus aculeatus microcephalus | Inland threespine stickleback | wide |
| Gasterosteus aculeatus williamsoni | Unarmored threespine stickleback | narrow |
| Siphatales mohavensis | Mojave tui chub | narrow |
| Siphatales bicolor pectinifer | Lahontan lake tui chub | narrow |
| Siphatales bicolor snyderi | Owens tui chub | narrow |
| Siphatales bicolor ssp. 1 | Eagle Lake tui chub | narrow |
| Siphatales thalassinus ssp. 1 | Pit River tui chub | wide |
| Siphatales thalassinus thalassinus | Goose Lake tui chub | narrow |
| Siphatales thalassinus vaccaceps | Cow Head tui chub | narrow |
| Hysterocarpus traskii lagunae | Clear Lake tule perch | narrow |
| Hysterocarpus traskii pomo | Russian River tule perch | narrow |
| Siphatales bicolor ssp. 11 | High Rock Spring Tui Chub | narrow |
| Cyprinodon nevadensis calidae | Tecopa Pupfish | narrow |
| Hysterocarpus traskii traskii | Sacramento tule perch | wide |
| Oncorhynchus mykiss - KMP winter | Klamath Mountains Province winter steelhead | anadromous |
| Oncorhynchus tshawytscha - SONCC fall | Southern Oregon Northern California coast fall Chinook salmon | anadromous |
| Lavinia exilicauda chi | Clear Lake hitch | narrow |
| Lavinia exilicauda exilicauda | Sacramento hitch | wide |
| Lavinia exilicauda harengeus | Monterey hitch | wide |
| Lavinia mitrulus | Northern (Pit) roach | narrow |
| Lavinia symmetricus navarroensis | Navarro roach | narrow |
| Lavinia parvipinnus | Gualala roach | narrow |
| Lavinia symmetricus ssp. 4 | Tomales roach | narrow |
| Lavinia symmetricus ssp. 2 | Red Hills roach | narrow |
| Lavinia symmetricus ssp. 3 | Clear Lake roach | narrow |
| Lavinia symmetricus subditus | Monterey roach | wide |
| Oncorhynchus clarki clarki | Coastal cutthroat trout | anadromous |
| Oncorhynchus clarki henshawi | Lahontan cutthroat trout | wide |
| Oncorhynchus clarki seleneris | Paiute cutthroat trout | narrow |
| Oncorhynchus kisutch - SONCC | Southern Oregon Northern California coast coho salmon | anadromous |
| Oncorhynchus kisutch - CCC | Central Coast coho salmon | anadromous |
| Oncorhynchus mykiss aguabonita | California golden trout | narrow |
| Oncorhynchus mykiss gilberti | Kern River rainbow trout | narrow |
| Oncorhynchus mykiss irideus | Coastal rainbow trout | wide |
| Oncorhynchus mykiss - Southern CA | Southern California steelhead | anadromous |
| Oncorhynchus mykiss - CV | Central Valley steelhead | anadromous |
| Oncorhynchus mykiss - KMP summer | Klamath Mountains Province summer steelhead | anadromous |
| Oncorhynchus mykiss aquilarum | Eagle Lake rainbow trout | narrow |
| Oncorhynchus mykiss ssp. 1 | Goose Lake redband trout | narrow |
| Oncorhynchus mykiss stonei | McCloud River redband trout | narrow |
| Oncorhynchus mykiss - CCC winter | Central California coast winter steelhead | anadromous |
| Oncorhynchus mykiss - SCCC | South Central California coast steelhead | anadromous |
| Oncorhynchus mykiss whitei | Little Kern golden trout | narrow |
| Oncorhynchus tshawytscha - CV spring | Central Valley spring Chinook salmon | anadromous |
| Oncorhynchus tshawytscha - CCC fall | California Coast fall Chinook salmon | anadromous |
| Oncorhynchus tshawytscha - CV winter | Central Valley winter Chinook salmon | anadromous |
| Rhinichthys osculus nevadensis | Amargosa Canyon speckled dace | narrow |
| Rhinichthys osculus ssp. 3 | Long Valley speckled dace | narrow |
| Rhinichthys osculus ssp. 2 | Owens speckled dace | narrow |
| Rhinichthys osculus ssp. 4 | Santa Ana speckled dace | narrow |
| Entosphenus tridentata ssp. 2 | Goose Lake lamprey | narrow |
| Acipenser medirostris ssp. 2 | Northern green sturgeon | anadromous |
| Acipenser transmontanus | White sturgeon | anadromous |
| Catostomus fumeiventris | Owens sucker | wide |
| Catostomus latipinnis | Flannelmouth sucker | narrow |
| Catostomus microps | Modoc sucker | narrow |
| Catostomus occidentalis occidentalis | Sacramento sucker | wide |
| Catostomus platyrhynchus | Lahontan mountain sucker | wide |
| Catostomus rimiculus | Klamath smallscale sucker | wide |
| Catostomus santaanae | Santa Ana sucker | wide |
| Catostomus snyderi | Klamath largescale sucker | wide |
| Catostomus tahoensis | Tahoe sucker | wide |
| Chasmistes brevirostris | Shortnose sucker | wide |
| Catostomus luxatus | Lost River sucker | wide |
| Xyrauchen texanus | Razorback sucker | wide |
| Siphatales bicolor bicolor | Klamath tui chub | narrow |
| Gila coerulea | Blue chub | narrow |
| Gila crassicauda | Thicktail Chub | wide |
| Gila elegans | Bonytail | wide |
| Gila orcutti | Arroyo chub | wide |
| Lavinia symmetricus symmetricus | Central California roach | wide |
| Mylopharodon conocephalus | Hardhead | wide |
| Orthodon microlepidotus | Sacramento blackfish | wide |
| Pogonichthys ciscoides | Clear Lake Splittail | narrow |
| Pogonichthys macrolepidotus | Sacramento splittail | wide |
| Ptychocheilus grandis | Sacramento pikeminnow | wide |
| Ptychocheilus lucius | Colorado Pikeminnow | wide |
| Richardsonius egregius | Lahontan redside | wide |
| Cyprinodon macularius | Desert pupfish | wide |
| Cyprinodon radiosus | Owens pupfish | narrow |
| Fundulus parvipinnis | California killifish | wide |
| Gasterosteus aculeatus aculeatus | Coastal threespine stickleback | wide |
| Hypomesus pacificus | Delta smelt | wide |
| Spirinchus thaleichthys | Longfin smelt | narrow |
| Thaleichthys pacificus | Eulachon | narrow |
| Archoplites interruptus | Sacramento perch | wide |
| Eucyclogobius newberryi | Tidewater goby | wide |
| Oncorhynchus gorbuscha | Pink salmon | anadromous |
| Oncorhynchus keta | Chum salmon | anadromous |
| Prosopium williamsoni | Mountain whitefish | wide |
| Salvelinus confluentus | Bull Trout | narrow |
| Cottus aleuticus | Coastrange sculpin | wide |
| Cottus asper ssp. 1 | Prickly sculpin | wide |
| Cottus asperrimus | Rough sculpin | narrow |
| Cottus beldingi | Paiute sculpin | wide |
| Cottus gulosus | Riffle sculpin | wide |
| Cottus pitensis | Pit sculpin | wide |
| Lampetra ayersi | River lamprey | anadromous |
| Entosphenus folletti | Northern California brook lamprey | narrow |
| Lampetra hubbsi | Kern brook lamprey | wide |
| Lampetra lethophaga | Pit-Klamath brook lamprey | wide |
| Lampetra richardsoni | Western brook lamprey | wide |
| Entosphenus similis | Klamath River lamprey | wide |
| Entosphenus tridentata ssp. 1 | Pacific lamprey | anadromous |
| Acipenser medirostris ssp. 1 | Southern green sturgeon | anadromous |
| Catostomus occidentalis humboldtianus | Humboldt sucker | wide |
| Catostomus occidentalis mnioltiltus | Monterey sucker | wide |
| Cottus asper ssp. 2 | Clear Lake prickly sculpin | narrow |
| Gasterosteus aculeatus ssp. 1 | Shay Creek stickleback | narrow |
| Lavinia symmetricus ssp. 1 | Russian River roach | wide |
| Oncorhynchus mykiss - NC winter | Northern California coast winter steelhead | anadromous |
| Oncorhynchus mykiss - NC summer | Northern California coast summer steelhead | anadromous |
| Oncorhynchus tshawytscha - UKT fall | Upper Klamath-Trinity fall Chinook salmon | anadromous |
| Oncorhynchus tshawytscha - UKT spring | Upper Klamath-Trinity spring Chinook salmon | anadromous |
| Oncorhynchus tshawytscha - CV late fall | Central Valley late fall Chinook salmon | anadromous |
| Oncorhynchus tshawytscha - CV fall | Central Valley fall Chinook salmon | anadromous |
| Rhinichthys osculus klamathensis | Klamath speckled dace | wide |
| Rhinichthys osculus robustus | Lahontan speckled dace | wide |
| Rhinichthys osculus ssp. 1 | Sacramento speckled dace | wide |
| Siphatales bicolor obesus | Lahontan stream tui chub | wide |
| Catostomus occidentalis lacusanserinus | Goose Lake sucker | narrow |
| Cottus klamathensis klamathensis | Upper Klamath marbled sculpin | narrow |
| Cottus klamathensis macrops | Bigeye marbled sculpin | narrow |
| Cottus klamathensis polyporus | Lower Klamath marbled sculpin | wide |
