# Supplementary material for: From fragmentation to resilience: Connectivity and habitat diversity as drivers of fish persistence in California watersheds
Source: PLoS One. 2025 Dec 23;20(12):e0339212. doi: 10.1371/journal.pone.0339212 (PMC12725570; doi:10.1371/journal.pone.0339212)
Supplement: S3 Table — Spearman correlation coefficients between HUC-12 TBI values and all HUC-12 physical habitat and condition variables by California freshwater ecoregion. Correlation p-value ≤0.001 = ***, ≤ 0.01 = **, ≤ 0.05 = *. Physical habitat variables with “km” are the percent change in length from current to historic while physical habitat variables without “km” are the change in presence/absence from current to historic. Condition variables are for current networks only. The first column for each ecoregion provides the correlation coefficient and p-value for the HUC-12s that had significant TBI values. The second column corresponds to all HUC-12 watersheds in each ecoregion. (DOCX) [file pone.0339212.s008.docx]

|  | **California Freshwater Ecoregion** | | | | | | | |
| --- | --- | --- | --- | --- | --- | --- | --- | --- |
|  | **Northern California** | | **Sacramento-San Joaquin** | | **Deserts-Lahontan** | | **Southern California** | |
| **Variable** | **Sig. TBI HUC12** | **All**  **HUC12s** | **Sig. TBI HUC12** | **All**  **HUC12s** | **Sig. TBI HUC12** | **All**  **HUC12s** | **Sig. TBI HUC12** | **All**  **HUC12s** |
| Stream & river km | -0.54*** | 0.03 | 0.06 | 0.08** | 0.71*** | -0.32*** | -0.24* | -0.5*** |
| Headwaters km | -0.53*** | -0.01 | 0.02 | 0.06* | 0.73*** | -0.32*** | -0.29* | -0.5*** |
| Creeks km | -0.48*** | 0.04 | 0.13 | 0.08** | 0.67*** | -0.3*** | -0.24* | -0.46*** |
| Small rivers km | -0.51*** | 0.06 | 0.15* | 0.08** | 0.58*** | -0.37*** | -0.16 | -0.39*** |
| Medium rivers km | -0.52*** | 0.13** | 0.12 | 0.12*** | 0.7*** | -0.31*** | 0.03 | -0.44*** |
| Mainstem km | -0.57*** | 0 | 0.14* | 0.16*** | 0.59*** | -0.19*** | -0.23 | -0.23*** |
| Large rivers km | -0.55*** | 0.04 | 0.15* | -0.02 | 0.64*** | -0.22*** | NA | -0.19*** |
| Great rivers km | -0.52*** | -0.42*** | -0.07 | 0.02 | 0.73*** | -0.36*** | NA | NA |
| Ocean/estuary connection km | -0.61*** | 0.03 | 0.15* | -0.01 | NA | NA | -0.01 | -0.41*** |
| Lake connection km | -0.46*** | 0.18*** | -0.24*** | 0.23*** | -0.22 | 0.01 | NA | NA |
| Artificial lake connection km | NA | NA | NA | NA | NA | NA | NA | NA |
| Very low gradient km | -0.57*** | 0.05 | 0.13 | 0.18*** | 0.76*** | -0.34*** | -0.14 | -0.43*** |
| Low gradient km | -0.59*** | 0.05 | 0.07 | 0.13*** | 0.59*** | -0.3*** | 0.02 | -0.43*** |
| Moderate gradient km | -0.62*** | 0.02 | 0.1 | 0.09*** | 0.43** | -0.32*** | 0.02 | -0.48*** |
| Moderate high gradient km | -0.47*** | 0.03 | 0.15* | 0.07** | 0.49*** | -0.27*** | -0.2 | -0.5*** |
| High gradient km | -0.48*** | 0.01 | 0.1 | 0.03 | 0.51*** | -0.37*** | -0.22 | -0.46*** |
| Steep gradient km | -0.5*** | -0.11* | 0.22** | 0.03 | 0.24 | -0.47*** | -0.26* | -0.37*** |
| Very cold temp km | -0.5*** | 0.04 | 0.29*** | -0.11*** | NA | 0.09 | NA | NA |
| Cold temp km | -0.49*** | 0.03 | 0.27*** | 0.03 | -0.58*** | -0.19*** | 0 | -0.14*** |
| Cool-cold temp km | -0.52*** | 0.05 | 0.18** | 0.04 | 0 | -0.44*** | -0.3** | -0.21*** |
| Cool temp km | -0.55*** | 0.05 | 0.02 | 0.06* | 0.31* | -0.36*** | -0.12 | -0.42*** |
| Cool-warm temp km | -0.63*** | 0.07 | 0.1 | 0.11*** | 0.47*** | -0.24*** | -0.02 | -0.56*** |
| Warm temp km | -0.51*** | 0.06 | 0.13 | 0.01 | 0.53*** | -0.22*** | 0.32** | -0.41*** |
| Confined km | -0.49*** | -0.12** | 0.07 | 0.05 | 0.24 | -0.45*** | -0.27* | -0.47*** |
| Moderately confined km | -0.54*** | 0.01 | 0.13 | 0.09*** | 0.71*** | -0.34*** | -0.06 | -0.49*** |
| Unconfined km | -0.64*** | 0.04 | 0.09 | 0.12*** | 0.54*** | -0.3*** | -0.1 | -0.46*** |
| Snowmelt flow km | -0.42*** | 0.05 | 0.14* | 0.07** | -0.2 | -0.05 | -0.02 | -0.38*** |
| Stable baseflow km | -0.56*** | 0.04 | 0.26*** | 0.05 | -0.16 | -0.07 | -0.07 | -0.33*** |
| Intermittent flow km | -0.5*** | 0.04 | 0.38*** | 0.05* | 0.62*** | -0.17** | 0.31** | -0.38*** |
| High elevation snowmelt km | NA | NA | 0.26*** | 0.04 | -0.14 | -0.1 | NA | NA |
| Intermittent SW flow km | -0.56*** | -0.06 | 0.03 | 0.08** | 0.51*** | -0.33*** | -0.16 | -0.49*** |
| Western runoff km | -0.45*** | -0.09* | 0.23** | 0.14*** | NA | NA | NA | -0.19*** |
| Habitat richness | -0.52*** | -0.2*** | 0.15* | 0.05 | 0.21 | -0.22*** | -0.28* | -0.52*** |
| Rare habitat richness | -0.6*** | 0.19*** | 0.13 | 0.1*** | 0.77*** | -0.25*** | -0.18 | -0.25*** |
| Headwaters | NA | 0 | 0.04 | -0.06* | NA | 0.03 | NA | -0.01 |
| Creeks | 0.1 | -0.04 | 0.04 | -0.06* | NA | NA | NA | -0.04 |
| Small rivers | 0.04 | -0.02 | -0.09 | 0 | 0.14 | -0.07 | -0.26* | -0.1* |
| Medium rivers | -0.21 | -0.32*** | -0.12 | 0.03 | 0.23 | -0.09 | -0.05 | -0.33*** |
| Mainstem | -0.59*** | -0.42*** | -0.03 | 0.05 | 0.68*** | -0.08 | -0.12 | -0.32*** |
| Large rivers | -0.61*** | -0.37*** | -0.04 | 0.02 | NA | NA | NA | -0.19*** |
| Great rivers | -0.52*** | -0.42*** | -0.07 | -0.02 | NA | -0.05 | NA | NA |
| Ocean/estuary connection | -0.62*** | -0.42*** | -0.07 | 0 | NA | NA | 0.02 | -0.4*** |
| Lake connection | -0.46*** | 0.18*** | -0.24*** | 0.22*** | NA | 0.01 | NA | NA |
| Artificial lake connection | 0.54*** | -0.07 | 0.05 | -0.14*** | -0.14 | -0.04 | -0.24* | 0.3*** |
| Very low gradient | NA | -0.03 | -0.06 | 0.04 | NA | 0.05 | -0.19 | -0.07 |
| Low gradient | -0.31* | -0.08 | -0.14* | -0.02 | NA | 0.1 | -0.17 | -0.18*** |
| Moderate gradient | 0.02 | -0.04 | -0.14 | -0.12*** | -0.01 | -0.04 | -0.11 | -0.11** |
| Moderate high gradient | 0.08 | -0.04 | -0.04 | -0.09*** | 0.16 | -0.04 | -0.34** | -0.18*** |
| High gradient | NA | -0.07 | 0.06 | -0.11*** | 0.2 | -0.05 | -0.03 | -0.08 |
| Steep gradient | 0.21 | -0.17*** | 0.03 | -0.05 | -0.19 | -0.25*** | -0.19 | -0.23*** |
| Very cold temp | -0.52*** | -0.42*** | 0.3*** | -0.15*** | NA | -0.05 | NA | NA |
| Cold temp | -0.03 | -0.18*** | 0.09 | -0.03 | -0.52*** | -0.16** | 0 | -0.33*** |
| Cool-cold temp | NA | 0.02 | 0 | -0.09*** | 0.2 | -0.03 | -0.16 | -0.31*** |
| Cool temp | -0.26* | -0.12** | -0.08 | -0.05* | 0.25 | -0.18*** | 0.16 | -0.15*** |
| Cool-warm temp | -0.68*** | -0.39*** | -0.23*** | 0.15*** | 0.14 | 0 | -0.23* | -0.22*** |
| Warm temp | -0.55*** | -0.39*** | -0.1 | 0.05 | -0.03 | -0.05 | 0.22 | -0.2*** |
| Confined | NA | 0.02 | 0.24*** | -0.1*** | NA | -0.06 | -0.14 | -0.11** |
| Moderately confined | 0.02 | 0 | 0.11 | 0.04 | NA | 0.03 | -0.13 | -0.08 |
| Unconfined | 0.02 | 0.01 | -0.11 | -0.03 | NA | 0.06 | -0.23 | -0.1* |
| Snowmelt flow | 0.04 | -0.03 | 0.06 | 0.04 | -0.01 | -0.03 | 0.06 | -0.24*** |
| Stable baseflow | -0.56*** | -0.31*** | 0.27*** | -0.09*** | NA | 0.05 | -0.11 | -0.33*** |
| Intermittent flow | -0.52*** | -0.42*** | 0.14* | 0.06* | 0.16 | 0.09 | 0.2 | -0.19*** |
| High elevation snowmelt | NA | NA | 0.26*** | 0.01 | NA | 0.09 | NA | NA |

**Table S-2 continued.**

|  | **California Freshwater Ecoregion** | | | | | | | |
| --- | --- | --- | --- | --- | --- | --- | --- | --- |
|  | **Northern California** | | **Sacramento-San Joaquin** | | **Deserts-Lahontan** | | **Southern California** | |
| **Variable** | **Sig. TBI HUC12** | **All**  **HUC12s** | **Sig. TBI HUC12** | **All**  **HUC12s** | **Sig. TBI HUC12** | **All**  **HUC12s** | **Sig. TBI HUC12** | **All**  **HUC12s** |
| Intermittent SW flow | -0.17 | -0.05 | -0.26*** | -0.08** | 0.35** | -0.03 | NA | NA |
| Western runoff | -0.04 | -0.28*** | 0.12 | 0.12*** | NA | NA | NA | -0.19*** |
| Dam storage | 0.56*** | -0.01 | -0.26*** | -0.1*** | -0.58*** | 0.01 | -0.1 | 0.19*** |
| Dam density | -0.14 | 0 | -0.28*** | -0.22*** | -0.62*** | 0.18*** | 0.53*** | 0.38*** |
| HWI | 0.36** | -0.16*** | 0.06 | -0.26*** | -0.16 | 0.09 | -0.21 | -0.03 |
| Historic habitat rarity | 0.49*** | -0.3*** | -0.16* | -0.13*** | -0.8*** | 0.35*** | 0.29* | 0.03 |
| Current habitat rarity | 0.12 | -0.45*** | -0.28*** | -0.15*** | -0.55*** | 0.26*** | -0.04 | -0.16*** |
| Habitat rarity change | -0.57*** | -0.38*** | -0.17* | -0.09*** | 0.45*** | -0.18*** | -0.09 | -0.14*** |
| % Habitat rarity change | -0.5*** | -0.41*** | -0.21** | 0.1*** | -0.06 | 0.15** | 0.36** | 0.09* |
| Local hydro regulation | 0.1 | 0.01 | 0.26*** | -0.39*** | 0.43** | -0.03 | 0.04 | 0.03 |
| Local water chemistry | 0.14 | 0.05 | 0.26*** | -0.42*** | 0.38** | 0.1 | -0.2 | -0.14*** |
| Local sediment regulation | 0.13 | -0.04 | 0.18* | -0.42*** | 0.34* | -0.1 | -0.06 | -0.03 |
| Local connectivity | 0.17 | -0.1* | 0.22** | -0.38*** | 0.47*** | 0.05 | -0.19 | -0.08* |
| Local temp regulation | 0.19 | -0.09* | 0.24*** | -0.39*** | 0.44*** | 0.04 | -0.18 | -0.08* |
| Local habitat provision | 0.1 | -0.1* | 0.24*** | -0.39*** | 0.37** | -0.09 | -0.17 | -0.08* |
| Local condition | 0.15 | -0.06 | 0.23*** | -0.41*** | 0.42** | -0.01 | -0.15 | -0.07 |
