# Supplementary material for: From fragmentation to resilience: Connectivity and habitat diversity as drivers of fish persistence in California watersheds
Source: PLoS One. 2025 Dec 23;20(12):e0339212. doi: 10.1371/journal.pone.0339212 (PMC12725570; doi:10.1371/journal.pone.0339212)
Supplement: S4 Table — Network variables by California freshwater ecoregion that were removed for the random forest analyses due to high correlation, multicollinearity, or zero variance. “Cor” indicates the variable exceeded the correlation threshold of 0.75. “VIF” identifies variables above the variance inflation threshold of 5. “ZV” refers to variables that had zero variance (i.e., values were identical for the ecoregion). Physical habitat variables with “km” are the percent change in length from current to historic while physical habitat variables without “km” are the change in presence/absence from current to historic. Condition variables are for current networks only. (DOCX) [file pone.0339212.s009.docx]

|  | **California Freshwater Ecoregion** | | | |
| --- | --- | --- | --- | --- |
| **Variable** | **Northern California** | **Sacramento-San Joaquin** | **Deserts-Lahontan** | **Southern California** |
| Pres/Abs of unconfined streams | Cor |  |  |  |
| Length (km) of unconfined streams | Cor | Cor | Cor | Cor |
| Length (km) of moderately confined streams | Cor | Cor | Cor | Cor |
| Presence/Absence of confined streams | Cor |  |  |  |
| Length (km) of confined streams | Cor | Cor | Cor | Cor |
| CCHEM_v2 Cemical component of the Index of Catchment Integrity (v2) from EPA StreamCat | Cor | Cor | Cor | Cor |
| CCONN_v2 - Hydrologic connectivity component score calculated using catchment metrics from EPA StreamCat | Cor | Cor | Cor | Cor |
| CHABT_v2 Habitat provision component score calculated using catchment metrics. | Cor | Cor | Cor | Cor |
| Richness (count) of habitat types present (based on presence-absence data) | Cor | Cor | VIF | Cor |
| CSED_v2 Sediment regulation component score calculated using catchment metrics. | Cor | Cor | Cor | Cor |
| CTEMP_v2 Temperature regulation component score calculated using catchment metrics. | Cor | Cor | Cor | Cor |
| Presence/Absence of creeks with drainage area of 10 to 100 km² |  |  | ZV |  |
| Length (km) of creeks with drainage area of 10 to 100 km² | Cor | Cor | Cor | Cor |
| Presence/absence of great rivers with drainage area > 25,000 km^2^ | Cor | Cor |  | ZV |
| Length (km) of great rivers with drainage area > 25,000 km^2^ | Cor | Cor | Cor | ZV |
| Length (km) of headwaters with drainage area of 2.5 to 10 km² | Cor | Cor | Cor | Cor |
| Pres/Abs of large rivers with drainage area of 10,000 to 25,000 km² | Cor | Cor | ZV | Cor |
| Length (km) of large rivers with drainage area of 10,000 to 25,000 km² | Cor |  |  |  |
| Length (km) of medium rivers with drainage area of 500 to 2500 km² | Cor | Cor | Cor | Cor |
| Pres/Abs of mainstem rivers with drainage area 2,500 to 10,000 km² | Cor |  | Cor | Cor |
| Length (km) of mainstem rivers with drainage area 2,500 to 10,000 km² | Cor | Cor | Cor |  |
| Presence/Absence of small rivers with drainage area of 100 to 500 km² |  |  | VIF |  |
| Length (km) of small rivers with drainage area of 100 to 500 km² | Cor | Cor | Cor | Cor |
| Volume of all reservoirs (NID_STORA in NID) per unit area of watershed (cubic meters/square km) from EPA StreamCat | Cor |  |  |  |
| Density of georeferenced dams within watershed (dams/ square km) based on the National Inventory of Dams (https://catalog.data.gov**) from EPA StreamCat** | ZV |  | ZV | ZV |
| Presence/Absence of streams with stable baseflow flow regime |  | Cor |  | Cor |
| Length (km) of streams with stable baseflow flow regime | Cor | Cor | Cor | Cor |
| Presence/Absence of streams high elevation snowmelt flow regime | ZV | Cor |  | ZV |
| Length (km) of streams high elevation snowmelt flow regime | ZV | Cor |  | ZV |
| Presence/Absence of streams with intermittent flow regime | Cor | Cor | VIF | Cor |
| Length (km) of streams with intermittent flow regime | Cor |  | Cor |  |
| Presence/Absence of streams with intermittent SW flow regime |  |  |  | ZV |
| Length (km) streams with intermittent SW flow regime | Cor | Cor | Cor | Cor |
| Length (km) of streams with snowmelt flow regime | Cor | Cor | Cor | VIF |
| Presence/Absence of streams with a Western runoff flow regime | Cor |  | ZV | Cor |
| Length (km) of streams with a Western runoff flow regime | Cor | Cor | ZV | Cor |
| Presence/Absence of an artificial lake | Cor |  |  |  |
| Length (km) of streams with access to an artificial lake | ZV | ZV | ZV | ZV |
| Presence/Absence of a natural lake | Cor | Cor | Cor | ZV |

| Length (km) of streams with access to a natural lake | Cor |  |  | ZV |
| --- | --- | --- | --- | --- |
| Pres/Abs of ocean or estuary | Cor | Cor | ZV | Cor |
| Length (km) of stream with access to an ocean or estuary | Cor | Cor | ZV | Cor |
| ICI_v2 Index of catchment integrity (v2) from EPA StreamCat | Cor | Cor | Cor | Cor |
| Rarity-weighted richness of habitat types present (p/a data) | Cor | Cor | Cor | VIF |
| Rarity-weighted richness of habitat types present (p/a data) for current networks | Cor |  | Cor | Cor |
| Rarity-weighted richness of habitat types present (p/a data) for historic networks | Cor | Cor | Cor |  |
| Z-score of Rarity-weighted richness of habitat types present (p/a data) of current networks | Cor | Cor | Cor | Cor |
| Z-score of Rarity-weighted richness of habitat types present (p/a data) of historic networks | Cor | Cor | Cor | Cor |
| Presence/Absence of streams with high gradient (4 – 10%) |  |  | VIF |  |
| Length (km) of streams with high gradient (4 – 10%) | Cor | Cor | Cor | Cor |
| Presence/Absence of streams with low gradient (0. 1 – 0. 5%) | VIF |  |  |  |
| Length (km) of streams with low gradient (0. 1 – 0. 5%) | Cor | Cor | Cor | Cor |
| Pres/Abs of streams with moderate gradient (0. 5 – 2%) | Cor |  |  |  |
| Length (km) of streams with moderate high gradient (2 - 4%) | Cor | Cor | Cor | Cor |
| Length (km) of streams with moderate gradient (0. 5 – 2%) | Cor | Cor | Cor | Cor |
| Length (km) of streams with steep gradient (> 10%) | Cor | Cor | Cor | Cor |
| Length (km) of streams with very low gradient (< 0. 1%) | Cor | Cor | Cor | Cor |
| Presence/Absenceof cool-cold (15 - 18°C) streams | Cor |  |  |  |
| Length (km) of cool-cold (15 - 18°C) streams | Cor | Cor | Cor |  |
| Presence/Absence of cold (10 – 15°C) streams | Cor |  |  | Cor |
| Length (km) of cold (10 – 15°C) streams | Cor | Cor | VIF |  |
| Length (km) of cool (18 - 21°C) streams | Cor | Cor | VIF | Cor |
| Presence/Absence of cool-warm (21 - 24°C) streams | Cor |  |  |  |
| Length (km) of cool-warm (21 - 24°C) streams | Cor | Cor | Cor | Cor |
| Total stream and river length in km |  | VIF |  |  |
| Presence/Absence of very cold (< 10°C) streams | Cor |  |  | ZV |
| Length (km) of very cold (< 10°C) streams | Cor | Cor | VIF | ZV |
| Pres/Abs of warm (> 24°C) streams | Cor | Cor | Cor | Cor |
| Length (km) of warm (> 24°C) streams | Cor | Cor | Cor |  |

**Table S-3 continued.**

|  | **Freshwater Ecoregion** | | | |
| --- | --- | --- | --- | --- |
| **Variable** | **Northern California** | **Sacramento-San Joaquin** | **Deserts-Lahontan** | **Southern California** |
| Change in z-score of rarity-weighted richness of habitat types between current and historic | Cor | Cor | Cor | VIF |
| Percent change in z-score of rarity-weighted richness of habitat types between current and historic | Cor | VIF |  |  |

*Model Performance*

After removing highly correlated and multicollinear variables, we built the non-spatial and spatial-RF models using the full dataset for each freshwater ecoregion. “Out-of-bag” (OOB) is the subset of the full dataset not used to build the component decision trees of each RF model. While OOB values are considered the best indicators of model performance, OOB data it not completely independent, especially if the data has high spatial autocorrelation. Table S-3 provides performance measures and the Moran’s I of the full non-spatial and spatial RF models for each freshwater ecoregion. A positive and significant Moran’s I value means that similar values are close to each other (clustering) while negative Moran’s I values indicate that data points in close proximity are more different. As Table S-3 shows, the inclusion of spatial predictors in the spatial RF models removed significant spatial autocorrelation in the RF model residuals and improved model performance in all freshwater ecoregions except the Deserts-Lahontan. Refer also to Figures S1-S4 to visualize Moran’s I plots the non-spatial and spatial RF models by ecoregion.
