# Supplementary material for: From fragmentation to resilience: Connectivity and habitat diversity as drivers of fish persistence in California watersheds
Source: PLoS One. 2025 Dec 23;20(12):e0339212. doi: 10.1371/journal.pone.0339212 (PMC12725570; doi:10.1371/journal.pone.0339212)
Supplement: S5 Table — Performance statistics (R2 and RMSE) for the full random forest models repeated 30 times for each California freshwater ecoregion. Out-of-bag (OOB) refers to the subset of the full dataset not used to build the individual decision trees of each RF model. The root mean squared error (RMSE) is the average difference between the actual values and those predicted by the RF model. The OOB values are the median and median absolute deviation (MAD) of the 30 model runs. Moran’s I is a measure of spatial autocorrelation with the median value for all distance thresholds reported in the table below. (DOCX) [file pone.0339212.s010.docx]

|  | **non-spatial RF** | | | **spatial RF** | | |
| --- | --- | --- | --- | --- | --- | --- |
| **Ecoregion** | **R^2^ (OOB)** | **RMSE (OOB)** | **Moran’s I**  **(p-value)** | **R^2^ (OOB)** | **RMSE (OOB)** | **Moran’s I**  **(p-value)** |
| Northern California | 0.668  +/- 0.0024 | 0.051  +/- 0.0004 | 0.087 (0) | 0.784  +/- 0.0032 | 0.041  +/- 0.0003 | -0.004 (0.641) |
| Sacramento-San Joaquin | 0.592  +/- 0.0026 | 0.117  +/- 0.0004 | 0.004 (0) | 0.786  +/- 0.0022 | 0.085  +/- 0.0004 | -0.00007 (0.352) |
| Deserts-Lahontan | 0.535  +/- 0.0025 | 0.186  +/-0.0005 | -0.001 (0.705) | 0.529  +/- 0.0037 | 0.187  +/- 0.0007 | -0.027 (.06) |
| Southern California | 0.62  +/- 0.0024 | 0.115  +/- 0.0004 | 0.011 (0) | 0.721  +/- 0.0028 | 0.098  +/- 0.0005 | 0.001 (0.15) |
