# Supplementary material for: From fragmentation to resilience: Connectivity and habitat diversity as drivers of fish persistence in California watersheds
Source: PLoS One. 2025 Dec 23;20(12):e0339212. doi: 10.1371/journal.pone.0339212 (PMC12725570; doi:10.1371/journal.pone.0339212)
Supplement: S6 Table — Rarity-weighted habitat richness values of historic and current networks were translated to z-scores by freshwater ecoregion and assigned to classes using these ranges. (DOCX) [file pone.0339212.s011.docx]

| Z-Score Class | Standard Deviation Range |
| --- | --- |
| Far Above Average | >2 |
| Above Average | >1 - ≤ 2 |
| Slightly Above Average | >0.5 - ≤ 1 |
| Average | > -0.5 - ≤ 0.5 |
| Slightly Below Average | > -1 - ≤ -0.5 |
| Below Average | >-2 - ≤ -1.0 |
| Far Below Average | ≤ -2 |
